# Evaluating the Performance of Mammogram-Based AI Risk Model in Predicting Subsequent Breast Cancer in Women with a Prior History of Breast Cancer

**DOI:** 10.3390/jcm15176507

**Published:** 2026-08-22

**Authors:** Samuel B. Ogunlade, Andrew Dakkak, Amie Leon, Kristin A. Robinson, Santo Maimone, Michael Villalba, Haley P. Letter

**Affiliations:** 1 Division of Interventional Radiology, Department of Radiology, Mayo Clinic, Jacksonville, FL 32224, USA; ogunlade.samuel@mayo.edu; 2 Division of Breast Imaging, Department of Radiology, Mayo Clinic, Jacksonville, FL 32224, USA; dakkak.andrew@mayo.edu (A.D.); leon.amie@mayo.edu (A.L.); robinson.kristin@mayo.edu (K.A.R.); maimone.santo@mayo.edu (S.M.); villalba.michael@mayo.edu (M.V.)

**Keywords:** subsequent breast cancer, ipsilateral recurrence, contralateral new primary breast cancer, artificial intelligence, mammogram, short-term risk prediction, machine learning

## Abstract

**Objectives:** Women with a history of breast cancer are at increased risk of developing subsequent breast cancer, including ipsilateral recurrence and contralateral new primary breast cancer. This study evaluated the discriminatory performance of a mammogram-based artificial intelligence (AI) risk model for predicting subsequent breast cancer within one year after a negative screening mammogram. **Methods:** This enriched retrospective case–control study included women with a prior history of breast cancer who underwent screening digital breast tomosynthesis between January 2018 and December 2023 at three affiliated academic breast imaging centers. Digital breast tomosynthesis examinations classified as BI-RADS 1 or 2 were retrospectively analyzed using the ProFound AI^®^ Risk model version 1.0 to estimate 1-year breast cancer risk. Patients were classified according to whether they developed subsequent breast cancer within one year of the index screening examination. Model discrimination was evaluated using receiver operating characteristic analysis. Sensitivity, specificity, positive predictive value, and negative predictive value were calculated at an exploratory cutoff selected by maximizing the Youden index. **Results:** The study included 96 women (mean age, 65.3 ± 8.7 years), of whom 32 developed subsequent breast cancer within one year, and 64 did not. The mean AI risk score was significantly higher in the subsequent breast cancer group than in the control group (1.18 ± 0.59 vs. 0.49 ± 0.41; *p* < 0.001). The AI model demonstrated an AUC of 0.824 (95% CI: 0.728–0.921). At an exploratory cutoff of 0.39, sensitivity was 81.3%, specificity was 76.6%, PPV was 63.4%, and NPV was 89.1%. In separate exploratory analyses, the AUC was 0.790 (95% CI: 0.641–0.939) for ipsilateral recurrence and 0.860 (95% CI: 0.752–0.974) for contralateral new primary breast cancer. AI risk scores were not significantly correlated with tumor size or age at subsequent breast cancer diagnosis. **Conclusions:** In this enriched retrospective case–control study, higher mammogram-based AI risk scores were associated with subsequent breast cancer within one year after a negative screening examination. The model demonstrated discriminatory performance for both ipsilateral recurrence and contralateral new primary breast cancer; however, these analyses were exploratory. Because the cohort was enriched for subsequent breast cancer events, the reported predictive values are specific to the study sample and should not be extrapolated to routine surveillance populations. Larger prospective cohorts are needed to validate discrimination, calibration, and clinical utility.

## 1. Introduction

Breast cancer remains the leading cause of cancer-related morbidity and mortality among women worldwide, with over 2 million new cases diagnosed annually [1]. Despite advancements in early detection, mammography continues to face limitations, particularly in detecting aggressive forms of cancer and identifying those at risk of subsequent breast cancer. False-negative results, where mammograms fail to detect existing cancers, pose a significant challenge, leading to delayed diagnoses and potentially poorer outcomes [2]. Interval cancers, those diagnosed after a negative mammogram and within the screening interval, account for a disproportionate number of breast cancer-related deaths and represent a major clinical concern [3].

The American Cancer Society (ACS) highlights that women with a history of breast cancer face an elevated risk of developing a subsequent breast cancer, including ipsilateral recurrence or contralateral new primary breast cancer, compared to the general population, and this increased risk is further supported by multiple meta-analyses [4,5,6]. The American College of Radiology (ACR) also recommends that such individuals begin Magnetic Resonance Imaging (MRI) screening at the earliest opportunity [7]. These guidelines stress the importance of close surveillance for this high-risk group. Despite these recommendations, the ability to predict which patients will develop subsequent breast cancer remains limited, and surveillance often relies on traditional imaging techniques and clinical judgment, which can be insufficient, particularly in detecting cancers that may appear between routine screenings. Furthermore, the identification of high-risk patients with a prior history of breast cancer is crucial, as they are at a significantly higher risk for recurrence compared to those with no previous history of breast cancer [8].

Recent advancements in artificial intelligence (AI) and machine learning offer the potential to overcome the challenges of risk assessment for women with a history of breast cancer. AI models trained on digital mammograms have demonstrated substantial promise in identifying subtle patterns within images that may indicate cancer, some of which may not be detectable by radiologists, offering an additional layer of analysis to refine existing risk assessments [9]. In fact, they have been shown to outperform traditional methods in predicting short-term breast cancer risk in high- and intermediate-risk individuals, and these models may enable earlier detection of malignancies, potentially reducing the incidence of interval cancers and improving patient outcomes [10,11,12,13,14]. More broadly, recent studies have highlighted the expanding role of AI and machine learning in individualized health-risk prediction across diverse clinical settings, supporting the development of personalized surveillance and risk-stratified care pathways [15,16,17].

However, while AI has proven effective in various high-risk groups, its performance in predicting subsequent breast cancer in women with a previous history of breast cancer remains largely unexplored. Given this critical need for more effective risk assessment strategies, this study aims to evaluate the ability of a mammogram-based AI risk model to predict subsequent breast cancer, including ipsilateral recurrence and contralateral new primary breast cancer, in a cohort of women with a history of breast cancer. 

## 2. Materials and Methods

### 2.1. Study Design and Setting

This retrospective observational study was approved by the Institutional Review Board of Mayo Clinic (No. 23-007303) and conducted in accordance with the Declaration of Helsinki and its subsequent amendments across three affiliated academic breast imaging centers of Mayo Clinic between January 2018 and December 2023. The study was approved under a waiver of informed consent due to its retrospective nature, and all procedures complied with the Health Insurance Portability and Accountability Act (HIPAA).

### 2.2. Study Population

The cohort consisted of women aged 18 years or older who underwent screening digital breast tomosynthesis (DBT) mammography during this time frame. Participants were divided into two groups: the cancer group, which included patients with a history of breast cancer who developed a subsequent breast cancer within one year of a non-actionable mammogram, and the control group, which consisted of women with a history of breast cancer but no subsequent breast cancer within one year following a non-actionable mammogram. The cancer group included patients who developed either ipsilateral breast cancer recurrence following breast-conserving therapy or subsequent new primary breast cancer in the ipsilateral or contralateral breast after the index screening mammogram. The primary study outcome was subsequent breast cancer diagnosed within one year of the index screening mammogram, including ipsilateral recurrence following breast-conserving therapy and contralateral new primary breast cancers. Throughout the manuscript, “subsequent breast cancer” refers to the composite outcome, whereas “recurrence” is reserved for ipsilateral events considered consistent with recurrence following breast-conserving therapy. Contralateral events are described as contralateral new primary breast cancers. To be eligible for inclusion, participants were required to have a previous history of breast cancer with no evidence of active or subsequent breast cancer at the time of the index mammogram, a negative screening mammogram classified as BI-RADS 1 or 2, performed between 2018 and 2023, and completed follow-up imaging and clinical records confirming the development or absence of subsequent breast cancer within one year of the index mammogram. Patients were excluded if they developed breast cancer beyond one year from the index mammogram or had missing follow-up data or incomplete imaging records.

All eligible patients who developed subsequent breast cancer within one year of the index mammogram were included. Potential controls were women with a prior history of breast cancer who had a BI-RADS 1 or 2 screening DBT examination during the same study period and no subsequent breast cancer during at least one year of documented imaging and clinical follow-up. Controls were randomly sampled in a 2:1 ratio, and their selection was performed without knowledge of imaging findings beyond the inclusion criteria. This approach was designed to balance analytic feasibility with statistical robustness and to demonstrate the feasibility of applying AI-based breast cancer risk assessment within a well-defined high-risk screening population, rather than to achieve a predetermined power calculation.

**Figure 1:**
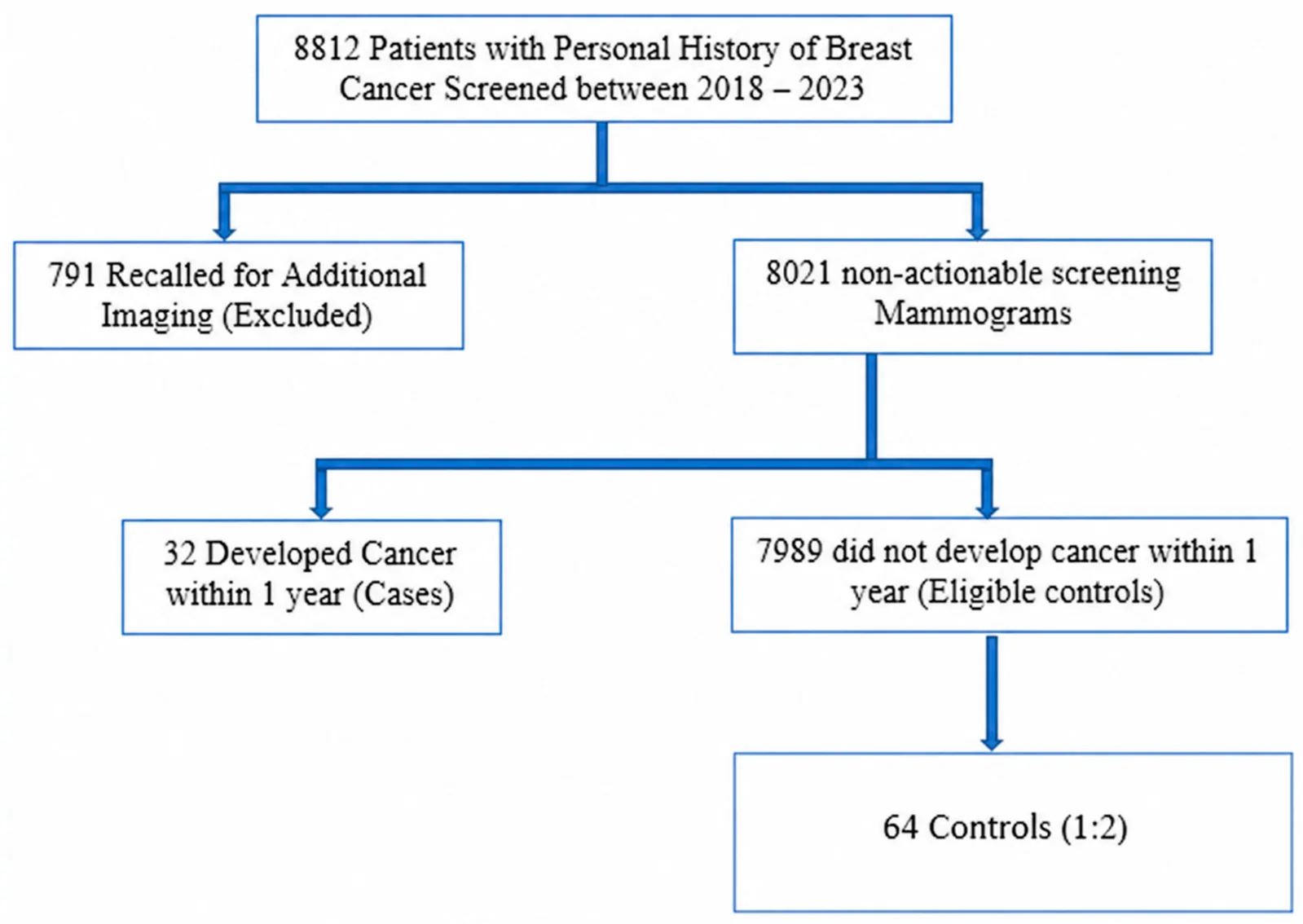
Study Population Selection Flowchart.

Given the relatively low incidence of subsequent breast cancer within one year after a negative screening mammogram, an enriched retrospective case–control design was used to evaluate model discrimination. All eligible subsequent breast cancer cases identified during the study period were included, and controls without subsequent breast cancer were selected at an approximate 2:1 control-to-case ratio. Because the proportion of patients with subsequent breast cancer was determined by the sampling design, the event prevalence in this cohort does not represent the prevalence in routine surveillance populations. Accordingly, the positive predictive value (PPV) and negative predictive value (NPV) are reported only as cohort-specific descriptive measures and should not be generalized to clinical screening populations. Given the exploratory objective of evaluating the discriminatory performance of the AI model and the relatively limited number of outcome events, multivariable regression analyses were not performed. The flowchart below (Figure 1) summarizes the entire selection process.

### 2.3. Imaging Protocols

#### 2.3.1. Digital Breast Tomosynthesis (DBT)

All patients underwent bilateral screening digital breast tomosynthesis (DBT) using Hologic systems (Hologic Inc., Marlborough, MA, USA). The examinations were interpreted by board-certified breast radiologists using the American College of Radiology Breast Imaging Reporting and Data System (ACR BI-RADS) lexicon. Only examinations initially classified as BI-RADS category 1 or 2, representing negative or benign findings, were included. All analyses were based on the original clinical interpretations made at the time of screening, without retrospective reinterpretation for the purposes of this study.

#### 2.3.2. Mammography-Based Artificial Intelligence (AI) Risk Model

The DBT examinations from all patients were retrospectively processed using the ProFound AI^®^ Risk model version 1.0 (iCAD Inc., Nashua, NH, USA). This image-based deep learning model was developed to estimate the 1-year risk of breast cancer following screening DBT. The model analyzes high-dimensional imaging features, including breast density, parenchymal texture, asymmetry, microcalcifications, and architectural distortion, using a multistage deep convolutional neural network.

The model was trained using a large, multi-institutional dataset independent of the cohort included in the present study. Accordingly, the current analysis represents an independent retrospective evaluation of the model’s predictive performance in women with a prior history of breast cancer.

The ProFound AI^®^ Risk model version 1.0 generates a continuous patient-level estimate of absolute 1-year breast cancer risk and classifies patients into four interpretive risk categories: low, general, moderate, and high risk. The continuous output is expressed as an estimated percentage risk of developing breast cancer within one year; for example, a score of 0.39 corresponds to an estimated 0.39% 1-year risk.

The risk estimate is derived from imaging and demographic features and is intended to quantify short-term breast cancer risk following a DBT screening examination. The model does not function as a lesion-detection or heatmap-based computer-aided detection system and does not provide breast-specific suspicious findings or lesion-localization outputs. It is distinct from the FDA-cleared ProFound AI^®^ Detection software, which is designed for lesion detection.

In this study, the ProFound AI^®^ Risk model version 1.0 was applied retrospectively for research purposes only and did not influence image interpretation, patient management, or clinical decision-making.

### 2.4. Data Analysis and Statistical Methods

Descriptive statistics were calculated for the cohort’s demographic, clinical, and imaging characteristics, stratified by subsequent breast cancer status (cancer group vs. control group). Continuous variables (age, AI risk scores, breast density scores) were reported as means with standard deviations and compared between groups using independent-samples *t*-tests. Categorical variables (menopausal status, family history, cancer subtype) were expressed as frequencies and percentages and compared using chi-squared tests. Breast density was categorized according to BI-RADS density categories (A–D). Differences in breast density distribution between patients with subsequent breast cancer and controls were assessed using the Pearson chi-squared test. Statistical significance was defined as a two-sided *p*-value < 0.05. Pearson’s correlation coefficient was used to assess the relationship between AI-assigned 1-year risk scores and tumor size. The diagnostic performance of the ProFound AI^®^ Risk model was assessed using Receiver Operating Characteristic (ROC) analysis, with calculation of the area under the curve (AUC). Sensitivity, specificity, PPV, and NPV were also calculated for the AI model. The cutoff was selected from the ROC curve by maximizing the Youden index, defined as sensitivity + specificity − 1, and should be regarded as exploratory. Because of the enriched case–control study design, PPV and NPV estimates should be interpreted within the context of the study cohort prevalence. Comparative performance across the AI model and other traditional risk models was visually summarized using forest plots of AUC values with 95% confidence intervals (CIs).

Subgroup analyses were performed to assess the consistency of the AI model’s performance across clinically relevant strata. These included analyses by tumor-related factors (histological subtype, grade, receptor status), menopausal status, laterality of the subsequent breast cancer event, and other factors. AI risk scores were compared between mass and non-mass enhancement lesions using the Mann–Whitney U test because of the small sample size and non-normal distribution. AI-assigned risk scores were also compared across subsequent breast cancer detection modality/context groups using the Kruskal–Wallis test due to small sample sizes and non-normality in several groups. AI risk scores were summarized by detection modality/context as medians with interquartile ranges (25th–75th percentile). A two-sided *p*-value <0.05 was considered statistically significant. All statistical analyses were conducted using IBM SPSS Statistics for Windows, Version 28.0 (IBM Corp., Armonk, NY, USA).

## 3. Results

### 3.1. Patient Baseline Characteristics and Features of the Previous Lesion

**Figure 2:**
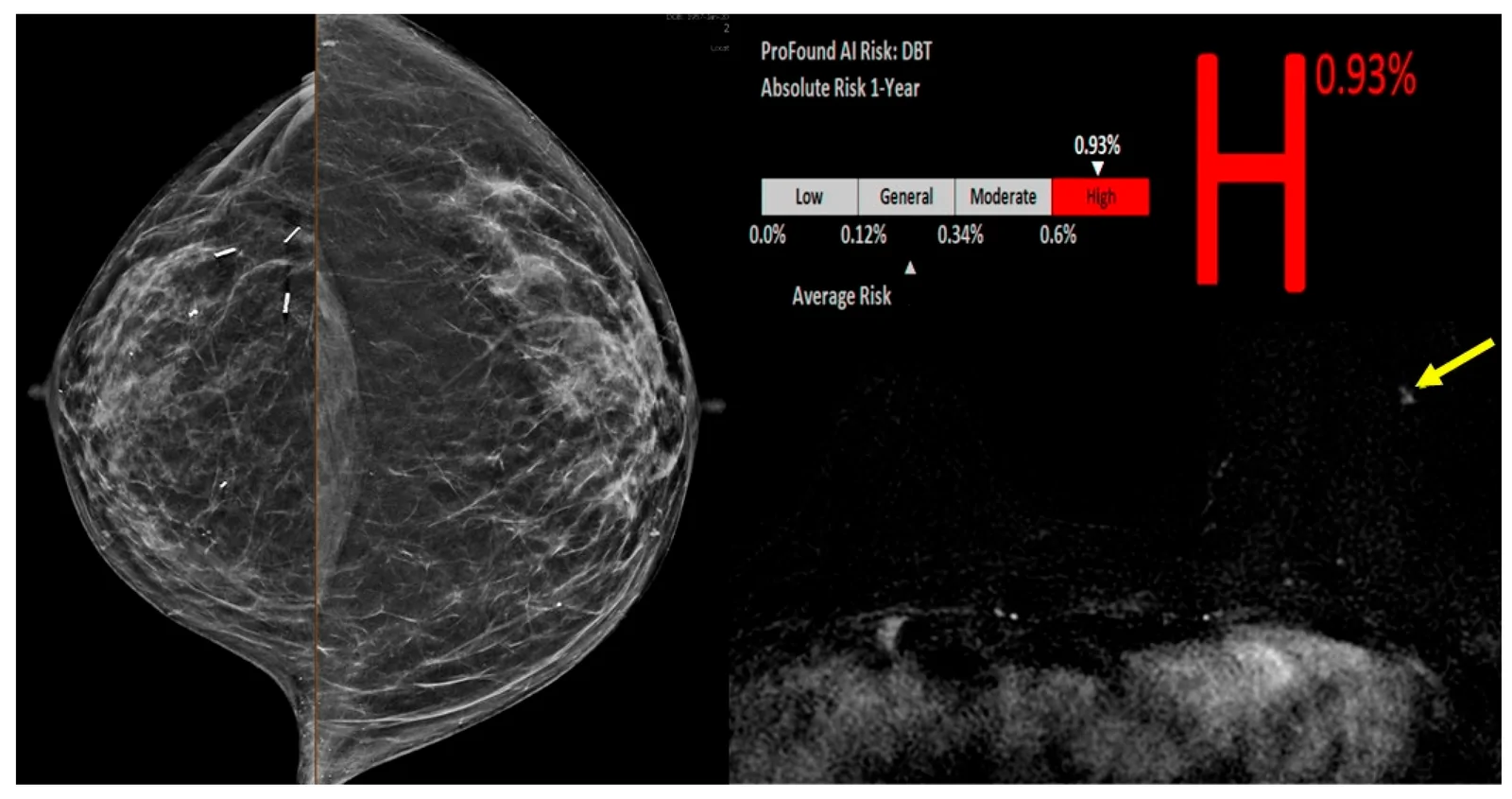
**(Subsequent breast cancer [contralateral new primary breast cancer]/Case):** 62-year-old BRCA 2 mutation carrier, with a history of right estrogen receptor–positive/progesterone receptor–positive IDC diagnosed 10 years prior, status post lumpectomy and adjuvant radiation. CC images from the index screening mammogram show heterogeneously dense tissue and lumpectomy changes in the right breast assessed as BI-RADS 2. The patient underwent high-risk screening breast MRI six months later, which showed focal non-mass enhancement lesion (yellow arrow) in the outer left breast assessed as BI- RADS 4. MRI-guided biopsy was performed, and pathology revealed DCIS. The AI risk score from the screening mammogram was high. The apparent difference in breast size reflects differences in patient positioning and compression during mammographic acquisition rather than a true anatomical difference.

**Figure 3:**
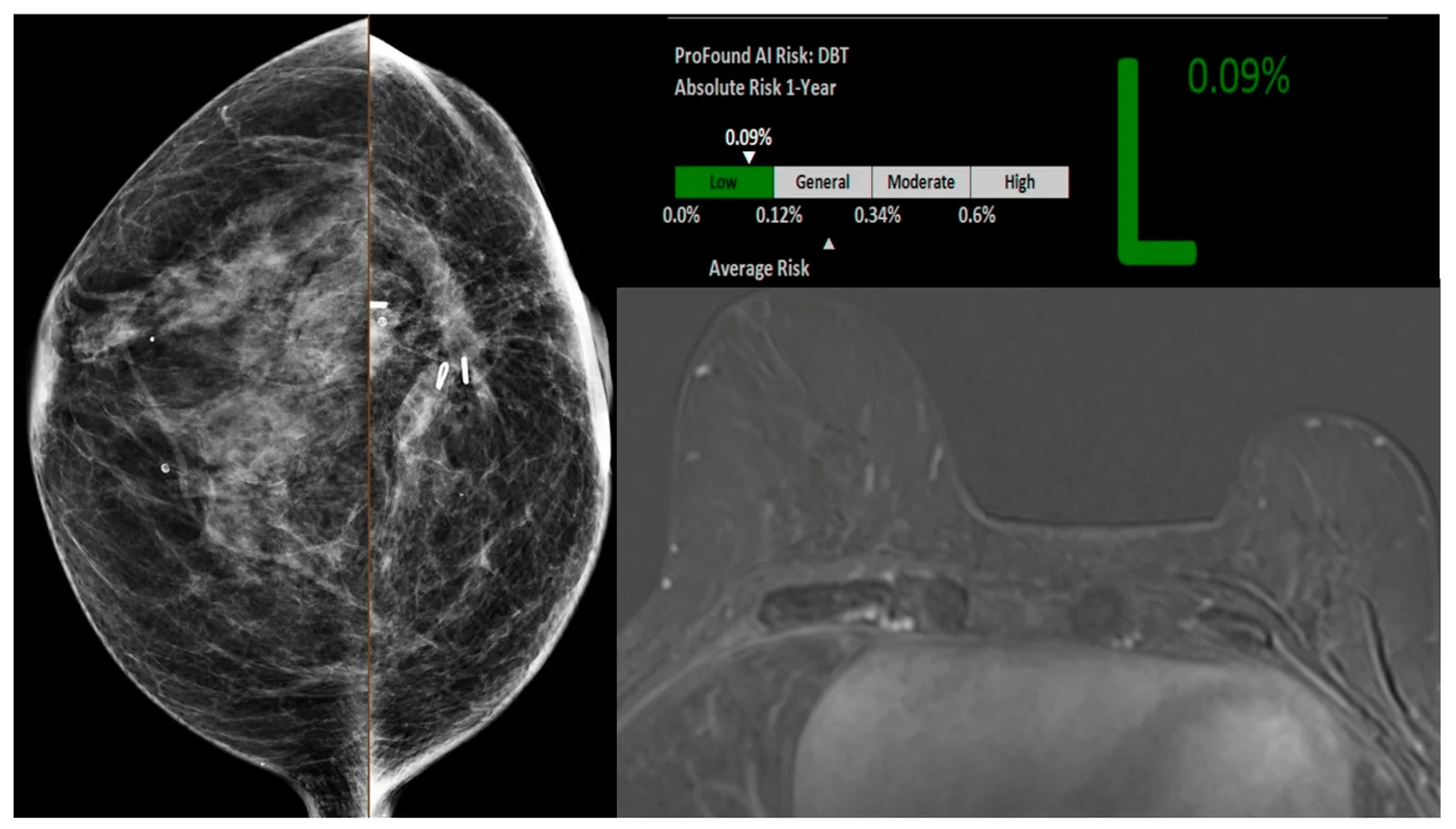
**(No subsequent breast cancer/Control):** 63-year-old with a history of left breast HER2-positive IDC 3 years prior, status post lumpectomy, adjuvant chemoradiation, and endocrine therapy. CC images from the index screening mammogram show heterogeneously dense tissue and lumpectomy changes in the left breast, assessed as BI-RADS 2. Patient underwent high-risk screening breast MRI, which was benign (BI-RADS 2). The AI risk score from the screening mammogram was low.

A total of 96 patients were included in this enriched study, with 32/96 (33.3%) developing subsequent breast cancer (Figure 2) and 64/96 (66.7%) remaining cancer-free within one year (Figure 3). The mean age was 65.31 ± 8.73 years, with no significant age difference between the two groups (*p* = 0.208). The majority of participants were White (89/96, 92.7%). The AI-assigned risk score was significantly higher in the subsequent breast cancer group (1.18 ± 0.59) compared with the control group (0.49 ± 0.41) (*p* < 0.001). There were no significant differences in age at first diagnosis (*p* = 0.186), largest tumor size (*p* = 0.863), histologic subtype (*p* = 0.924), grade (*p* = 0.604), or race (*p* = 0.937). Most participants were postmenopausal (88.5%), and the majority had IDC (67.7%). Endocrine therapy was more commonly administered to patients without subsequent breast cancer (73.4%) than to patients with subsequent breast cancer (43.8%) (*p* = 0.011). Other characteristics of the study population and features of the previous lesion are detailed in Table 1.

### 3.2. Characteristics of the Subsequent Breast Cancer Group

In the subsequent breast cancer group, the time from initial breast cancer diagnosis to the subsequent breast cancer event was fairly evenly distributed, with the majority (9/32, 28.1%) developing subsequent breast cancer 8–12 years after the initial diagnosis. In addition, 18/32 (56.2%) developed contralateral new primary breast cancer, and 8/32 (25.0%) had DCIS. Tumor size at the subsequent breast cancer diagnosis was most commonly in the 7–12 mm range (10/32, 31.2%). Grade 2 tumors were the most prevalent (17/32, 53.1%). ER and PR positivity were found in 24/32 (75.0%) of patients. HER2 was negative in 23/32 (71.9%), and 22/32 (68.8%) had negative nodal status. The majority of subsequent breast cancers were detected by screening MRI (16/32, 50.0%), and AI-assigned risk scores did not differ significantly by the modality/context in which the subsequent breast cancer was detected (Kruskal–Wallis χ^2^ = 1.597, *p* = 0.809). No significant difference in breast density distribution was observed between groups (χ^2^ = 1.03, *p* = 0.793). Further details are summarized in Table 2, Table 3 and Table 4 below. When compared with controls without subsequent breast cancer, AI risk scores were significantly higher among both ipsilateral recurrence cases (0.97 ± 0.42 vs. 0.49 ± 0.41, *p* < 0.001) and contralateral new primary breast cancer cases (1.22 ± 0.66 vs. 0.49 ± 0.41, *p* < 0.001) (Table 5).

### 3.3. Correlation Between AI Risk Scores and Clinical Factors

Table 6 presents the correlations between AI risk scores and key clinical factors, including tumor size, age at subsequent breast cancer diagnosis, time from initial cancer diagnosis to subsequent breast cancer event, and age at first diagnosis. The correlation coefficients (Pearson’s r) indicate weak, non-significant relationships for all factors: AI risk score and largest tumor size (r = −0.036, *p* = 0.845), AI risk score and age at subsequent breast cancer diagnosis (r = 0.070, *p* = 0.705), and AI risk score and the time from initial cancer diagnosis to subsequent breast cancer event (r = −0.125, *p* = 0.495). Similarly, AI risk scores showed a weak correlation with age at first diagnosis (r = 0.165, *p* = 0.368).

### 3.4. Diagnostic Performance of the AI Risk Model

The AI model demonstrated strong discriminative performance for predicting subsequent breast cancer with an AUC of 0.824 (95% CI: 0.728–0.921, *p* < 0.001). The model achieved a sensitivity of 81.3%, specificity of 76.6%, PPV of 63.4%, and NPV of 89.1% (Table 7).

### 3.5. Performance of the AI Model Across Various Subgroups

Exploratory subgroup analyses yielded AUC estimates ranging from 0.786 to 0.870. Overall, the estimates were broadly similar across subgroups but imprecise, with wide confidence intervals in several categories due to small subgroup sample sizes (Table 8).

### 3.6. AI Risk Scores and MRI Lesion Type

Among the patients whose subsequent breast cancer was detected through MRI, there was no significant difference in AI risk scores between those with mass lesions (median = 1.18) and those with non-mass lesions (median = 0.96) (*p* = 0.71) (Table 9).

## 4. Discussion

In this study, an AI-driven model stratified the risk of subsequent breast cancer, distinguishing those who developed subsequent breast cancer from controls. Traditional clinical and histopathologic factors did not significantly differ between groups, whereas AI risk scores were significantly higher in the subsequent breast cancer group.

Although tumor size and grade correlate with subsequent breast cancer in large studies, conventional predictors, including age, histology, receptor status, and nodal involvement, often provide limited individualized prediction during long-term survivorship monitoring [18]. The lack of significant differences in traditional factors suggests that the risk of subsequent breast cancer may not be fully captured by standard clinical variables. Subsequent breast cancer development may be shaped by microenvironmental interactions, host immunity, tumor dormancy, and subclonal genomic evolution, which staging and histopathology cannot fully capture [19,20]. Identical stages may yield divergent outcomes due to molecular and phenotypic dynamics [21,22]. Conversely, AI risk scores differentiated patients with and without subsequent breast cancer, suggesting that they may indicate the presence of latent imaging biomarkers.

The lack of significant differences in AI-assigned risk scores according to subsequent cancer detection modality or context suggests that the AI model may capture an underlying risk of subsequent breast cancer that is independent of the eventual method of detection. This lack of significant differences should, however, be interpreted cautiously, given the small sample sizes in several detection categories, which limited statistical power. Nevertheless, these findings support the potential utility of AI-based risk assessment as a modality-independent marker of subsequent breast cancer risk and warrant validation in larger multi-institutional cohorts.

The key finding is that AI assigned higher risk scores to patients who later developed subsequent breast cancer, suggesting that imaging contains clinically meaningful signals preceding these events. Prior studies show that deep learning mammographic models can predict future breast cancer risk, especially in high-risk individuals, and Ogunlade et al. reported similar findings in intermediate-risk patients undergoing supplemental molecular breast imaging [10,12,13,14]. These data support the presence of latent parenchymal and structural imaging signals linked to early carcinogenesis. Dembrower et al. further showed that AI mammographic models improve risk prediction by capturing parenchymal complexity and tissue heterogeneity [23]. Similarly, radiomic models based on preoperative multiparametric MRI and imaging-derived texture and shape features have predicted disease-free survival and recurrence more accurately than clinicopathologic nomograms [24,25]. Unlike tissue-based genomic assays such as Oncotype DX^®^ or MammaPrint^®^, which require tissue sampling and are costly, AI imaging models are non-invasive and repeatable for surveillance [26,27]. Deep learning using MRI and whole-slide histology has also outperformed clinical nomograms and gene-expression assays [28,29].

AI systems may analyze high-dimensional imaging patterns that conventional models cannot capture, including microarchitecture, signal intensity, lesion boundaries, and parenchymal interactions. These phenotypes may reflect angiogenesis, stromal remodeling, immune infiltration, hypoxia, and early tumor evolution that are linked to recurrence and survival but are not visible to humans [30,31]. The limited associations between the AI score and the evaluated clinical variables suggest that it may provide orthogonal risk information, consistent with radiomics studies [32,33]. Breast density is an established breast cancer risk factor and an important imaging characteristic that can influence both cancer detection and risk prediction models [34,35]. The lack of significant differences in breast density distribution between the two groups in this study suggests that breast density was well balanced between groups and is unlikely to have substantially confounded the observed association between AI-derived mammographic risk scores and subsequent breast cancer.

AI performance across receptor subtypes, including ER-positive and HER2-negative disease, although currently exploratory, may be clinically relevant, as these subtypes may influence timing and biology. Hormone receptor-positive tumors often recur later, while triple-negative and HER2-positive tumors recur earlier and more aggressively [15,19]. AI features may reflect subtype-related microenvironments. MRI radiomics correlate with PAM50 subtypes, proliferation, and recurrence-related gene expression, supporting prediction beyond receptor status alone [36,37].

More than half of the subsequent breast cancer events occurred contralaterally, with the highest mean AI risk scores also in contralateral cancers, consistent with elevated second-primary risk among survivors from shared genetic, environmental, and treatment-related factors [5,6,38]. The predominance of intermediate-grade and ER-positive events among patients with subsequent breast cancer may partly reflect the persistent long-term risk associated with hormone receptor–positive disease [15]. The lower frequency of endocrine therapy in the subsequent breast cancer group is consistent with Pan et al., who showed that 5 years of endocrine therapy reduces recurrence, although distant recurrence risk persists after cessation, especially among patients with higher-grade or node-positive disease [39]. Because the present composite outcome also included contralateral new primary breast cancers, this association should not be interpreted as evidence of a recurrence-specific effect. These findings suggest that AI-derived risk scores may, in the near future, eventually help identify patients who could benefit from individualized surveillance strategies; however, prospective validation is required before clinical implementation.

AI risk scores did not differ between mass and NME lesions among MRI-detected subsequent breast cancers, suggesting that the model may capture both structural abnormalities and broader breast cancer risk–related imaging signatures. This is clinically relevant because NME can be visually challenging and may reflect diffuse tissue changes preceding a breast cancer diagnosis [40]. The lack of correlation between AI scores and tumor size, time to the subsequent breast cancer event, or age further supports the possibility of independent risk prediction from latent imaging features, such as texture, rather than solely the detection of existing tumors. AI scores could potentially complement clinical models in hybrid frameworks, as shown in radiomics studies [41,42].

The enriched case–control design facilitated evaluation of model discrimination but precluded estimation of population-level absolute risk and clinically generalizable predictive values. Consequently, PPV and NPV were strongly influenced by the sampling design and should not be interpreted as population-level estimates. In a clinical population with a substantially lower annual event rate, the PPV would be expected to be considerably lower, even if sensitivity and specificity remained unchanged. The present study therefore evaluates model discrimination rather than real-world risk stratification. Also, due to the enriched case–control design, calibration metrics, including calibration plots, calibration intercept, calibration slope, and Brier score, as well as decision curve analysis, would also not provide clinically meaningful estimates. Consequently, the model’s calibration and its net clinical benefit across different clinical decision thresholds remain unknown. Future prospective cohort studies with representative real-world event rates are needed to evaluate calibration, decision-curve analysis, and the incremental clinical utility of AI-assisted risk assessment.

### Limitations and Future Directions

Although the findings are encouraging, some limitations warrant consideration. The modest overall sample size and limited racial diversity may restrict generalizability. Future longitudinal studies involving larger, multi-institutional, racially and ethnically diverse cohorts will be essential to validate performance and ensure broad applicability, as genetic and environmental factors may influence recurrence risk and treatment effectiveness.

Additionally, while exploratory subgroup analyses yielded encouraging AUC estimates, the results should be interpreted as exploratory given the small sample sizes within strata, and external prospective validation is critical before clinical adoption. Further research should also explore multimodal integration, combining AI imaging signatures with genomic, molecular, and proteomic data, as well as digital pathology data, to enhance predictive accuracy, as recent advances in genomics have revealed that genetic mutations, epigenetic alterations, and tumor microenvironment characteristics play a significant role in breast cancer outcomes [43]. Techniques such as federated learning and transfer learning could facilitate cross-institutional training without compromising patient privacy [44].

Another limitation is that multivariable analyses were not performed to evaluate whether the AI risk score provides predictive information independent of established clinical factors. Consequently, residual confounding cannot be excluded, particularly given the observed difference in endocrine therapy use between the groups that had subsequent breast cancer and those who did not. The present study was designed primarily to evaluate the AI model’s discriminative performance rather than to establish its independent prognostic value. Larger prospective studies with sufficient numbers of outcome events should incorporate multivariable modeling to determine the incremental value of mammogram-based AI risk assessment beyond conventional clinicopathologic risk factors.

Moreover, the cutoff of 0.39 was selected by maximizing the Youden index in the same dataset used to evaluate model performance. Consequently, the associated sensitivity, specificity, PPV, and NPV may be optimistically biased, and this exploratory threshold requires validation in an independent cohort before clinical application.

Furthermore, longitudinal modeling that incorporates temporal changes in imaging and treatment response trajectories may capture dynamic risk patterns more effectively than static models. The calibration and decision-analytic performance of the model were not formally assessed in this study, highlighting key areas for future research.

## 5. Conclusions

In this enriched retrospective study of women with a prior history of breast cancer and a negative screening mammogram, higher AI risk scores were associated with subsequent breast cancer diagnosed within one year. Higher AI risk scores were observed in both ipsilateral recurrence and contralateral new primary breast cancer groups, although these subgroup findings were exploratory. The study does not establish population-level predictive values and is insufficient to support the use of the model in clinical surveillance. Larger, diverse, prospective cohorts are needed to validate discrimination, assess calibration and clinical utility, evaluate the two outcome types separately, and determine whether AI risk scores provide information beyond established clinical and treatment-related factors.

## Figures and Tables

**Table 1:** Baseline characteristics of the study population and previous breast cancer.

Characteristics	Overall (N = 96)	No Subsequent Breast Cancer (Control Group, N = 64)	Subsequent Breast Cancer (Case Group, N = 32)	*p*-Value
**Age (mean ± SD)**	65.31 ± 8.73	64.52 ± 9.30	66.91 ± 7.32	0.208
**AI Risk Score (mean ± SD)**	0.72 ± 0.54	0.49 ± 0.41	1.18 ± 0.59	<0.001
**Age at First Diagnosis (mean ± SD)**	53.78 ± 8.95	52.94 ± 8.82	55.50 ± 9.09	0.186
**Largest Tumor Size (mm) (mean ± SD)**	15.98 ± 9.25	16.10 ± 9.21	15.75 ± 9.47	0.863
**Race**				0.937
White	89 (92.7%)	59 (92.2%)	30 (93.8%)	
Black	3 (3.1%)	2 (3.1%)	1 (3.1%)	
Asian	4 (4.2%)	3 (4.7%)	1 (3.1%)	
**Ethnicity**				0.975
Hispanic	2 (2.1%)	1 (1.6%)	1 (3.1%)	
Non-Hispanic	94 (97.9%)	63 (98.4%)	31 (96.9%)	
**Menopausal Status**				0.257
Postmenopausal	85 (88.5%)	55 (85.9%)	30 (93.8%)	
Premenopausal	11 (11.5%)	9 (14.1%)	2 (6.3%)	
**Laterality**				0.128
Left	52 (54.2%)	35 (54.7%)	17 (53.1%)	
Right	42 (43.8%)	29 (45.3%)	13 (40.6%)	
Bilateral	2 (2.1%)	0 (0.0%)	2 (6.3%)	
**Histology**				0.924
DCIS	23 (24.0%)	16 (25.0%)	7 (21.9%)	
IDC	65 (67.7%)	43 (67.2%)	22 (68.8%)	
ILC	8 (8.3%)	5 (7.8%)	3 (9.4%)	
**Grade**				0.604
Grade 1	24 (25.0%)	18 (28.1%)	6 (18.8%)	
Grade 2	41 (42.7%)	26 (40.6%)	15 (46.9%)	
Grade 3	31 (32.3%)	20 (31.3%)	11 (34.4%)	
**ER Status**				0.170
Positive	74 (77.1%)	52 (81.3%)	22 (68.8%)	
Negative	22 (22.9%)	12 (18.8%)	10 (31.3%)	
**PR Status**				0.773
Positive	71 (74.0%)	48 (75.0%)	23 (71.9%)	
Negative	25 (26.0%)	16 (25.0%)	9 (28.1%)	
**HER2 Status**				0.989
Positive	15 (15.6%)	10 (15.6%)	5 (15.6%)	
Negative	81 (84.4%)	54 (84.4%)	27 (84.4%)	
**Nodal Status**				0.491
Positive	34 (35.4%)	24 (37.5%)	10 (31.3%)	
Negative	56 (58.3%)	35 (54.7%)	21 (65.6%)	
None	6 (6.3%)	5 (7.8%)	1 (3.1%)	
**Surgery Type**				0.527
Lumpectomy	84 (87.5%)	57 (89.1%)	27 (84.4%)	
Mastectomy	12 (12.5%)	7 (10.9%)	5 (15.6%)	
**Radiation**				0.727
Yes	86 (89.6%)	58 (90.6%)	28 (87.5%)	
No	10 (10.4%)	6 (9.4%)	4 (12.5%)	
**Chemotherapy**				0.520
Yes	44 (45.8%)	31 (48.4%)	13 (40.6%)	
No	52 (54.2%)	33 (51.6%)	19 (59.4%)	
**Endocrine Therapy**				0.011
Yes	61 (63.5%)	47 (73.4%)	14 (43.8%)	
No	35 (36.5%)	17 (26.6%)	18 (56.2%)	
**Genetic Testing**				0.957
Yes	36 (37.5%)	23 (35.9%)	13 (40.6%)	
No	60 (62.5%)	41 (64.1%)	19 (59.4%)	
**Genetic Testing Result**				0.458
Negative	29 (80.6%)	20 (87.0%)	9 (69.2%)	
BRCA1	2 (5.6%)	1 (4.3%)	1 (7.7%)	
BRCA2	1 (2.8%)	0 (0.0%)	1 (7.7%)	
Other Pathogenic Variants	4 (11.1%)	2 (8.7%)	2 (15.4%)	

**Table 2:** Characteristics of the Subsequent Breast Cancer Group (N = 32).

Characteristics	n	Percentage
**Time from initial cancer diagnosis to subsequent breast cancer (Years)**		
0–3	8	25.0%
4–7	8	25.0%
8–12	9	28.1%
13–18	5	15.6%
19–30	2	6.3%
**Laterality of subsequent breast cancer**		
Ipsilateral recurrence	14	43.8%
Contralateral new primary breast cancer	18	56.2%
**Histology of subsequent breast cancer**		
DCIS	8	25.0%
IDC	18	56.2%
ILC	6	18.8%
**Tumor size of subsequent breast cancer (mm)**		
2–6	6	18.8%
7–12	10	31.2%
13–18	7	21.9%
19–33	9	28.1%
**Grade of subsequent breast cancer**		
1	8	25.0%
2	17	53.1%
3	7	21.9%
**ER of subsequent breast cancer**		
Positive	24	75.0%
Negative	8	25.0%
**PR of subsequent breast cancer**		
Positive	24	75.0%
Negative	8	25.0%
**HER2 of subsequent breast cancer**		
Positive	1	3.1%
Negative	23	71.9%
Unknown/Not reported	8	25.0%
**Nodal Status of subsequent breast cancer**		
Positive	2	6.2%
Negative	22	68.8%
None	8	25.0%
**Detection Context and Modality**		
Screening MRI	16	50.0%
Self-Examination	3	9.4%
Incidental on Chest CT	3	9.4%
MRI for Implant Integrity	1	3.1%
MRI Lumbar Spine for Back Pain	1	3.1%
Screening Mammography	7	21.9%
Screening MBI	1	3.1%
**MRI Feature**		
Mass	22	68.8%
Non-mass	10	31.2%

**Table 3:** Breast Density Distribution Between Patients with Subsequent Breast Cancer and Controls.

Breast Density	Controls (n = 64)	Subsequent Breast Cancer (n = 32)	*p*-Value
**A**	1 (1.6%)	0 (0.0%)	0.793
**B**	18 (28.1%)	11 (34.4%)	
**C**	39 (60.9%)	19 (59.4%)	
**D**	6 (9.4%)	2 (6.3%)	

**Table 4:** AI-assigned risk scores according to Subsequent Breast Cancer detection modality/context.

Detection Modality/Context	N (32)	Median AI Score (IQR: Q1–Q3)	*p*-Value *
Screening MRI	16	0.96 (0.39–1.60)	0.809
Self-examination	3	0.60 (0.52–NR)	
Incidental on chest CT	3	0.93 (0.23–NR)	
Screening mammography	7	1.29 (0.88–1.81)	
Other imaging modalities ^†^	3	1.21 (0.09–NR)	

* Kruskal–Wallis test. NB: Values are presented as median (25th–75th percentile). NR = not reliably estimable because of the small sample size within the group. ^†^ Includes MRI performed for implant integrity (n = 1), MRI of the lumbar spine for back pain (n = 1), and screening molecular breast imaging (MBI) (n = 1). NR = Not reliably estimable because of the small sample size.

**Table 5:** AI-assigned risk scores according to recurrence laterality.

Group	N	Mean AI Risk Score ± SD	*p*-Value vs. Control *
Controls (No subsequent breast cancer)	64	0.49 ± 0.41	Reference
Ipsilateral recurrence	14	0.97 ± 0.42	<0.001
Contralateral new primary breast cancer	18	1.22 ± 0.66	<0.001

* vs. control.

**Table 6:** Correlation Between AI Risk Scores and Tumor Size, Age at Subsequent Breast Cancer, Time Between Initial Breast Cancer and Subsequent Breast Cancer, and Age at First Diagnosis.

		Largest Tumor Size	Age at Subsequent Breast Cancer Diagnosis (Years)	Time from Initial Cancer Diagnosis to Subsequent Breast Cancer (Years)	Age at First Diagnosis (Years)
**AI risk score**	Pearson Correlation (r)	−0.036	0.070	−0.125	0.165
	*p*-value	0.845	0.705	0.495	0.368
	N	32	32	32	32

**Table 7:** Discriminative Performance of the AI Risk Model for Predicting Subsequent, Ipsilateral Recurrence and Contralateral New Breast Cancer among Patients with a History of Breast Cancer.

Model	AUC (95% CI)	*p*	Optimal Cutoff	Sensitivity	Specificity	PPV	NPV
Subsequent breast cancer	0.824 (0.728–0.921)	<0.001	0.39	81.3%	76.6%	63.4%	89.1%
Ipsilateral recurrence	0.790 (0.641–0.939)	0.02	0.42	78.6%	76.6%	42.3%	94.2%
Contralateral new primary	0.860 (0.752–0.974)	0.003	0.48	83.3%	78.1%	51.7%	94.3%

**Table 8:** Performance Of The AI Model Across Various Subgroups for Predicting Subsequent Breast Cancer.

*# Subgroup	Cases	Control	AUC (95% CI)	*p*
**Menopausal Stage**				
Premenopausal	2	9	0.789 (0.380–0.982)	<0.001
Postmenopausal	30	55	0.840 (0.744–0.936)	<0.001
**Tumor Type**				
IDC (Invasive Ductal Carcinoma)	22	43	0.818 (0.701–0.936)	<0.001
ILC (Invasive Lobular Carcinoma)	3	5	0.825 (0.486–0.998)	<0.001
DCIS (Ductal Carcinoma In Situ)	7	16	0.838 (0.637–0.973)	<0.001
**Tumor Grade**				
Grade 1	6	18	0.870 (0.675–0.992)	<0.001
Grade 2	15	26	0.823 (0.679–0.966)	<0.001
Grade 3	11	20	0.800 (0.624–0.976)	<0.001
**Receptor Status**				
ER Positive	22	52	0.836 (0.724–0.948)	<0.001
ER Negative	10	12	0.786 (0.586–0.986)	0.003
PR Positive	23	48	0.821 (0.707–0.935)	<0.001
PR Negative	9	16	0.828 (0.643–0.981)	<0.001
HER2 Negative	27	54	0.822 (0.717–0.927)	<0.001
HER2 Positive	5	10	0.790 (0.521–0.982)	<0.001

* All subgroup analyses were exploratory because of the small sample sizes within each subgroup and should be interpreted cautiously. # Subgroup analysis was not done separately for the ipsilateral and contralateral due to the extremely small sample size for each of them.

**Table 9:** AI Risk Scores and MRI Lesion Type Among Patients with MRI-Detected Subsequent Breast Cancer (N = 16).

Lesion Type	n (%)	Median AI Score (IQR)	*p*-Value
Mass	11 (68.8)	1.18 (0.89–1.63)	
NME	5 (31.2)	0.96 (0.60–1.45)	0.71

## Data Availability

The data presented in this study are not publicly available because they contain protected patient health information and are subject to institutional privacy and ethical restrictions. De-identified data may be available from the corresponding author upon reasonable request and with permission from the Mayo Clinic Institutional Review Board, where applicable.

## Abbreviations

The following abbreviations are used in this manuscript:

ACR	American College of Radiology
ACS	American Cancer Society
AI	Artificial Intelligence
AUC	Area Under the Receiver Operating Characteristic Curve
BI-RADS	Breast Imaging Reporting and Data System
BRCA1	Breast Cancer Gene 1
BRCA2	Breast Cancer Gene 2
CAD	Computer-Aided Detection
CC	Craniocaudal (mammographic view)
CI	Confidence Interval
CNN	Convolutional Neural Network
CT	Computed Tomography
DBT	Digital Breast Tomosynthesis
DCIS	Ductal Carcinoma In Situ
ER	Estrogen Receptor
HER2	Human Epidermal Growth Factor Receptor 2
HIPAA	Health Insurance Portability and Accountability Act
IBM SPSS	IBM Statistical Package for the Social Sciences
IDC	Invasive Ductal Carcinoma
ILC	Invasive Lobular Carcinoma
IQR	Interquartile Range
LR+	Positive Likelihood Ratio
LR−	Negative Likelihood Ratio
MBI	Molecular Breast Imaging
MRI	Magnetic Resonance Imaging
NME	Non-Mass Enhancement
NPV	Negative Predictive Value
NR	Not Reliably Estimable
PAM50	Prediction Analysis of Microarray 50-gene breast cancer subtype classifier
PPV	Positive Predictive Value
PR	Progesterone Receptor
Q1	First Quartile
Q3	Third Quartile
ROC	Receiver Operating Characteristic
SD	Standard Deviation
USA	United States of America
WISDOM	Women Informed to Screen Depending On Measures of Risk (breast cancer screening trial)

## References

[1] World Health Organization (2025). Breast Cancer: Key Facts [Internet].

[2] Oluyemi E.T., Grimm L.J., Goldman L., Lee M., Pittman S.M., Rosen E., Zuley M.L., Lee C.S. (2026). False-Negative Screening and Diagnostic Mammograms in the National Mammography Database From 2010 to 2022. AJR Am. J. Roentgenol..

[3] Niraula S., Biswanger N., Hu P., Lambert P., Decker K. (2020). Incidence, Characteristics, and Outcomes of Interval Breast Cancers Compared with Screening-Detected Breast Cancers. JAMA Netw. Open.

[4] Sung H., Freedman R.A., Siegel R.L., Hyun N., DeSantis C.E., Ruddy K.J., Jemal A. (2021). Risks of subsequent primary cancers among breast cancer survivors according to hormone receptor status. Cancer.

[5] Parhizgar P., Bahadori Monfared A., Mohseny M., Keramatinia A., Hashemi Nazari S.S., Rahman S.A., Al Marzouqi A., Al-Yateem N., Mosavi Jarrahi A. (2023). Risk of second primary cancer among breast cancer patients: A systematic review and meta-analysis. Front. Oncol..

[6] Molina-Montes E., Requena M., Sánchez-Cantalejo E., Fernández M.F., Arroyo-Morales M., Espín J., Arrebola J.P., Sánchez M.J. (2015). Risk of second cancers cancer after a first primary breast cancer: A systematic review and meta-analysis. Gynecol. Oncol..

[7] Monticciolo D.L., Newell M.S., Moy L., Niell B., Monsees B., Sickles E.A. (2018). Breast Cancer Screening in Women at Higher-Than-Average Risk: Recommendations From the ACR. J. Am. Coll. Radiol..

[8] Martei Y.M., Matro J.M. (2015). Identifying patients at high risk of breast cancer recurrence: Strategies to improve patient outcomes. Breast Cancer (Dove Med. Press).

[9] McKinney S.M., Sieniek M., Godbole V., Godwin J., Antropova N., Ashrafian H., Back T., Chesus M., Corrado G.S., Darzi A. (2020). International evaluation of an AI system for breast cancer screening. Nature.

[10] Yala A., Lehman C., Schuster T., Portnoi T., Barzilay R. (2019). A Deep Learning Mammography-based Model for Improved Breast Cancer Risk Prediction. Radiology.

[11] Pike J., Hoffmeister J. (2023). Estimating the Likelihood of Near-Term Breast Cancer Using ProFound AI^®^ Risk: Theory and Clinical Performance [White Paper].

[12] Eriksson M., Destounis S., Czene K., Zeiberg A., Day R., Conant E.F., Schilling K., Hall P. (2022). A risk model for digital breast tomosynthesis to predict breast cancer and guide clinical care. Sci. Transl. Med..

[13] Eriksson M., Román M., Gräwingholt A., Castells X., Nitrosi A., Pattacini P., Heywang-Köbrunner S., Rossi P.G. (2023). European validation of an image-derived AI-based short-term risk model for individualized breast cancer screening-a nested case-control study. Lancet Reg. Health Eur..

[14] Ogunlade S.B., Wang L., Maimone S., Robinson K.A., Moran K.M., Leon A., Morozov A.P., Nwachukwu C.T., Letter H.P. (2026). Mammogram-based AI risk assessment in patients with dense breasts undergoing supplemental molecular breast imaging. Quant. Imaging Med. Surg..

[15] Du J., Zhu L., Min X., Chang X., Qi W., Wei T., Wei S., Zhang X., Huang J., Tao X. (2026). A study on promoting AI learning and usage behaviors among health management students from the perspective of the “knowledge-belief-action” model. Front. Public Health.

[16] Feng X., Zhu X., Peng J., Huang Y., Huang H., Tan Y., Li X. (2026). Developing and evaluating machine learning-based risk models for metabolic syndrome among nurses: A cross-sectional study. Front. Public Health.

[17] Ji Q., Wang M. (2026). AI-driven high-risk pregnancy prediction: Balancing early detection, anxiety, and discrimination in digital public health. Front. Public Health.

[18] Waks A.G., Winer E.P. (2019). Breast Cancer Treatment: A Review. JAMA.

[19] Sosa M.S., Bragado P., Aguirre-Ghiso J.A. (2014). Mechanisms of disseminated cancer cell dormancy: An awakening field. Nat. Rev. Cancer.

[20] Tufail M., Jiang C.H., Li N. (2025). Tumor dormancy and relapse: Understanding the molecular mechanisms of cancer recurrence. Mil. Med. Res..

[21] Harbeck N., Penault-Llorca F., Cortes J., Gnant M., Houssami N., Poortmans P., Ruddy K., Tsang J., Cardoso F. (2019). Breast cancer. Nat. Rev. Dis. Prim..

[22] Yates L.R., Knappskog S., Wedge D., Farmery J.H.R., Gonzalez S., Martincorena I., Alexandrov L.B., Van Loo P., Haugland H.K., Lilleng P.K. (2017). Genomic Evolution of Breast Cancer Metastasis and Relapse. Cancer Cell.

[23] Dembrower K., Liu Y., Azizpour H., Eklund M., Smith K., Lindholm P., Strand F. (2020). Comparison of a Deep Learning Risk Score and Standard Mammographic Density Score for Breast Cancer Risk Prediction. Radiology.

[24] Liu Z., Li Z., Qu J., Zhang R., Zhou X., Li L., Sun K., Tang Z., Jiang H., Li H. (2019). Radiomics of Multiparametric MRI for Pretreatment Prediction of Pathologic Complete Response to Neoadjuvant Chemotherapy in Breast Cancer: A Multicenter Study. Clin. Cancer Res..

[25] Fan W., Cui H., Liu X., Zhang X., Fang X., Wang J., Qin Z., Yang X., Tian J., Zhang L. (2025). Machine learning-based ultrasound radiomics for predicting risk of recurrence in breast cancer. Front. Oncol..

[26] Hsieh T.Y., Huang C.C., Tseng L.M. (2025). Multi-gene expression assays in breast cancer: A literature review. Transl. Cancer Res..

[27] de Jongh F.E., Efe R., Herrmann K.H., Spoorendonk J.A. (2022). Cost and Clinical Benefits Associated with Oncotype DX® Test in Patients with Early-Stage HR+/HER2- Node-Negative Breast Cancer in the Netherlands. Int. J. Breast Cancer.

[28] Choi S., Lee Y., Lee M., Byon J.H., Choi E.J. (2025). Deep Learning-Based Recurrence Prediction in HER2-Low Breast Cancer: Comparison of MRI-Alone, Clinicopathologic-Alone, and Combined Models. Diagnostics.

[29] Cruz-Roa A., Gilmore H., Basavanhally A., Feldman M., Ganesan S., Shih N.N.C., Tomaszewski J., González F.A., Madabhushi A. (2017). Accurate and reproducible invasive breast cancer detection in whole-slide images: A Deep Learning approach for quantifying tumor extent. Sci. Rep..

[30] Dercle L., McGale J., Zhao B., Schmitt J., Peltzer A., Schwartz L.H., Amend M. (2025). Artificial intelligence and radiomics biomarkers for treatment response prediction in advanced HER2-negative breast cancer. Breast.

[31] Wu J., Li B., Sun X., Cao G., Rubin D.L., Napel S., Ikeda D.M., Kurian A.W., Li R. (2017). Heterogeneous Enhancement Patterns of Tumor-adjacent Parenchyma at MR Imaging Are Associated with Dysregulated Signaling Pathways and Poor Survival in Breast Cancer. Radiology.

[32] Lambin P., Leijenaar R.T.H., Deist T.M., Peerlings J., de Jong E.E.C., van Timmeren J., Sanduleanu S., Larue R.T.H.M., Even A.J.G., Jochems A. (2017). Radiomics: The bridge between medical imaging and personalized medicine. Nat. Rev. Clin. Oncol..

[33] Kumar V., Gu Y., Basu S., Berglund A., Eschrich S.A., Schabath M.B., Forster K., Aerts H.J., Dekker A., Fenstermacher D. (2012). Radiomics: The process and the challenges. Magn. Reson. Imaging.

[34] Boyd N.F., Guo H., Martin L.J., Sun L., Stone J., Fishell E., Jong R.A., Hislop G., Chiarelli A., Minkin S. (2007). Mammographic density and the risk and detection of breast cancer. N. Engl. J. Med..

[35] Tice J.A., Miglioretti D.L., Li C.S., Vachon C.M., Gard C.C., Kerlikowske K. (2015). Breast Density and Benign Breast Disease: Risk Assessment to Identify Women at High Risk of Breast Cancer. J. Clin. Oncol..

[36] Mazurowski M.A., Zhang J., Grimm L.J., Yoon S.C., Silber J.I. (2014). Radiogenomic analysis of breast cancer: Luminal B molecular subtype is associated with enhancement dynamics at MR imaging. Radiology.

[37] Zhu Y., Li H., Guo W., Drukker K., Lan L., Giger M.L., Ji Y. (2015). Deciphering Genomic Underpinnings of Quantitative MRI-based Radiomic Phenotypes of Invasive Breast Carcinoma. Sci. Rep..

[38] Kurian A.W., McClure L.A., John E.M., Horn-Ross P.L., Ford J.M., Clarke C.A. (2009). Second primary breast cancer occurrence according to hormone receptor status. J. Natl. Cancer Inst..

[39] Pan H., Gray R., Braybrooke J., Davies C., Taylor C., McGale P., Peto R., Pritchard K.I., Bergh J., Dowsett M. (2017). 20-Year Risks of Breast-Cancer Recurrence after Stopping Endocrine Therapy at 5 Years. N. Engl. J. Med..

[40] Cheng L., Li X. (2012). Breast Imaging Reporting and DataSystem (BI-RADS) of magnetics resonance imaging: Breast mass. Gland Surg..

[41] Tsai H.Y., Tsai T.Y., Wu C.H., Chung W.S., Wang J.C., Hsu J.S., Hou M.F., Chou M.C. (2022). Integration of Clinical and CT-Based Radiomic Features for Pretreatment Prediction of Pathologic Complete Response to Neoadjuvant Systemic Therapy in Breast Cancer. Cancers.

[42] Li H., Zhu Y., Burnside E.S., Drukker K., Hoadley K.A., Fan C., Conzen S.D., Whitman G.J., Sutton E.J., Net J.M. (2016). MR Imaging Radiomics Signatures for Predicting the Risk of Breast Cancer Recurrence as Given by Research Versions of MammaPrint, Oncotype DX, and PAM50 Gene Assays. Radiology.

[43] Coyle K.M., Boudreau J.E., Marcato P. (2017). Genetic Mutations and Epigenetic Modifications: Driving Cancer and Informing Precision Medicine. BioMed Res. Int..

[44] Sheller M.J., Edwards B., Reina G.A., Martin J., Pati S., Kotrotsou A., Milchenko M., Xu W., Marcus D., Colen R.R. (2020). Federated learning in medicine: Facilitating multi-institutional collaborations without sharing patient data. Sci. Rep..

